# Acetaminophen Mitigates Myocardial Injury Induced by Lower Extremity Ischemia-Reperfusion in Rat Model

**DOI:** 10.21470/1678-9741-2017-0218

**Published:** 2018

**Authors:** Onur Geldi, Emre Kubat, Celal Selçuk Ünal, Suat Canbaz

**Affiliations:** 1 Department of Cardiovascular Surgery, Zonguldak Atatürk State Hospital, Zonguldak, Turkey.; 2 Department of Cardiovascular Surgery, Karabük Training and Research Hospital, Karabük, Turkey.; 3 Department of Cardiovascular Surgery, Trakya University Faculty of Medicine, Edirne, Turkey.

**Keywords:** Aorta, Abdominal, Acetaminophen, Reperfusion Injury

## Abstract

**Objective:**

The injury-reducing effect of acetaminophen, an effective analgesic and antipyretic on ischemia-reperfusion continues to attract great attention. This study analyzed the protective effect of acetaminophen on myocardial injury induced by ischemia-reperfusion in an experimental animal model from lower extremity ischemia-reperfusion.

**Methods:**

Twenty-four Sprague-Dawley female rats were randomized into three groups (n=8) as (i) control group (only laparotomy), (ii) aortic ischemia-reperfusion group (60 min of ischemia and 120 min of reperfusion) and (iii) ischemia-reperfusion + acetaminophen group (15 mg/kg/h intravenous acetaminophen infusion starting 15 minutes before the end of the ischemic period and lasting till the end of the reperfusion period). Sternotomy was performed in all groups at the end of the reperfusion period and the heart was removed for histopathological examination. The removed hearts were histopathologically investigated for myocytolysis, polymorphonuclear leukocyte (PMNL) infiltration, myofibrillar edema and focal hemorrhage.

**Results:**

The results of histopathological examination showed that acetaminophen was detected to particularly diminish focal hemorrhage and myofibrillar edema in the ischemia-reperfusion + acetaminophen group (*P*<0.001, *P*=0.011), while there were no effects on myocytolysis and PMNL infiltration between the groups (*P*=1.000, *P*=0.124).

**Conclusion:**

Acetaminophen is considered to have cardioprotective effect in rats, by reducing myocardial injury induced by abdominal aortic ischemia-reperfusion.

**Table:** 

Abbreviations, acronyms & symbols	
APAP	= N-acetyl-p-aminophenol
PMNL	= Polymorphonuclear leukocyte

## INTRODUCTION

Ischemia is the shortage of oxygen and metabolites needed for continuity of cellular metabolism, resulting from the restriction of blood supply to tissues. Timely blood flow to ischemic tissue limits ischemia only to reversible cell damage. Blood re-flow to the ischemic tissue is important for the prevention of irreversible tissue damage; however, reperfusion may increase tissue damage caused by ischemia. This is defined as "ischemia-reperfusion injury" and induces a wide pathologic spectrum from reversible cell damage to multi-organ dysfunction^[^^1^^]^. Several studies are ongoing to halt or slow the effects of ischemia-reperfusion injury with preconditioning or postconditioning, while there are also molecular studies to reduce or halt the activity of oxygen radicals^[^^2^^-^^4^^]^. Many previous studies have reported the presence of reactive oxygen radicals resulting from cross-clamping to abdominal aorta, and myocardial ischemia-reperfusion injury induced by inflammatory response in the surgical treatment of abdominal aortic aneurysm, which is a common disease in cardiovascular surgery^[^^4^^-^^6^^]^.

Increase in cardiac morbidity and mortality and, in turn, cardioprotective approaches, have been subject to many studies to date. Therapeutic studies particularly aimed at the reduction and prevention of cardiac ischemia-reperfusion injury still continue to be relevant^[^^7^^-^^9^^]^. Acetaminophen or N-acetyl-p-aminophenol (APAP), which is included in the list of essential medicines for the primary health system, published by the World Health Organization, is commonly used as an analgesic and antipyretic^[^^10^^]^. APAP has been used in some experimental studies as a cardioprotective agent during myocardial ischemia-reperfusion^[^^11^^-^^13^^]^. There is no histopathological study in the literature addressing the cardioprotective role of APAP in cardiac distant organ ischemia-reperfusion injury induced by lower extremity ischemia. In this light, the present study was aimed to make histopathological examination of whether acetaminophen, which has well-defined analgesic and antipyretic effects and which is currently addressed by laboratory and pre-clinical studies for its cardioprotective effects, reduced the myocardial ischemia-reperfusion injury induced by lower extremity ischemia induced by cross-clamping to the abdominal aorta.

## METHODS

## Animals and Experimental Groups

This study was conducted after receiving approval (Protocol No: TÜHDYEK-2011/70 and Decision No: 2011.09.04) of the Trakya University Animal Experiments Local Ethics Committee. Experimental animals were obtained from the Experimental Animals Unit of the Faculty of Medicine, Trakya University. Twenty-four female Sprague-Dawley rats approximately 3-5 months old and weighing 190-250 grams were randomized in equal numbers (n=8) into the control group, the ischemia-reperfusion (I/R) group and the ischemia-reperfusion + acetaminophen (I/R+A) group. Rats were kept in rooms automatically illuminated for 12 hours and darkened for the subsequent 12 hours, heated at 20-22ºC and moisturized by 40-45% during the whole experiment. During this period, all rats were kept in transparent cages and fed with standard rat food, and tap water.

The rats were randomized into three groups (n=8) as (i) control group (only laparotomy), (ii) aortic ischemia-reperfusion group (60 min of ischemia and 120 min of reperfusion), and (iii) ischemia-reperfusion + acetaminophen group (15 mg/kg/h intravenous acetaminophen infusion starting 15 minutes before the end of the ischemic period and lasting till the end of the reperfusion period). In a study, Kiris et al.^[^^4^^]^ found that cardiac free oxygen radicals production, lipid peroxidation, and neutrophil activation significantly increased after 30 min ischemia and 60 min reperfusion in rats undergoing cross-clamping to the abdominal aorta. Therefore, in our study, we aimed to perform a histopathological examination after applying myocardial ischemia-reperfusion injury through 60 min ischemia and 120 min reperfusion in a rat model.

## Surgical Technique

All study rats were weighted before the procedures and their weights were recorded. After eight hours of fasting, ketamine hydrochloride 40 mg/kg (Ketalar® 50 mg/ml flacon, Pfizer) + xylazine hydrochloride 5 mg/kg (Rompun® 23.32 mg/ml, 50 ml flacon, Bayer) were administered intramuscularly under anesthesia.

An additional dose of ketamine hydrochloride was planned to be administered if required during the experiment. An additional dose of ketamine hydrochloride was administered to ensure spontaneous respiration of rats during the whole procedure. Rats were laid on the table in the supine position under a heating lamp. Samples were cannulated in the tail vein, using a yellow cannula. After the skin of all rats be prepared aseptically, laparotomy was performed through the midline just under the xiphoid process up to 0.5 cm above the pubis. The intestines were shifted to the right using a wet cloth after the laparotomy. The infrarenal abdominal aorta was explored via blunt dissection. All rats were administered heparin (100 unit/kg) (Nevparin® 25000 IU 5 ml flacon, Mustafa Nevzat) as an anticoagulant. 10 ml/kg 0.9% NaCl was administered from the tail vein for fluid resuscitation during the whole experiment. Infusion was performed using the Braun® Perfusor.

### Ischemia-Reperfusion (I/R) Group

An atraumatic microvascular clamp (Novaclip® 12 mm Angle) was inserted in the infrarenal abdominal aorta. After clamping, approximately 5 ml of warm physiological saline solution was administered to the peritoneal cavity. The abdomen was closed by three silk sutures to prevent fluid loss. Following 60 min ischemia, the atraumatic microvascular clamp was removed from the infrarenal abdominal aorta and a 120 min of reperfusion period was introduced. Aortic ischemia was followed by pulsation loss after the clamping procedure and reperfusion by aorta pulsation after clamp removal. After these procedures, the rats were sacrificed and their hearts removed.

### Ischemia-Reperfusion + Acetaminophen (I/R+A) Group

In addition to the procedures performed on the rats in the ischemia-reperfusion group, rats in this group were started on infusion with 15 mg/kg/min acetaminophen (Perfalgan 1000 mg/100 ml flacon, Bristol-Myers Squibb) 15 minutes before the aortic clamp was removed, with the infusion lasting till the end of the reperfusion period. Infusion was performed through the tail vein using Braun® Perfusor. At the end of the procedures, the rats were sacrificed and their hearts removed.

### Control Group

No clamping was performed in the aorta. 5 ml of warm, 0.9% NaCl was injected in the abdomen which was then closed by three silk sutures to prevent fluid loss. The rats were kept under this condition for 180 minutes, the time equivalent to 60 min of ischemia plus 120 min of reperfusion performed in the other two groups. The rats were then sacrificed and their hearts removed.

## Histopathological Examination

Sternotomy was performed in all groups at the end of the reperfusion period, and the heart was removed for histopathological examination. The tissues were fixed in 10% neutral-buffered formalin (Carlo Erba Reagents) and embedded in paraffin wax. Ten-micrometer - thick serial sections were obtained and stained with hematoxylin and eosin (Sigma Aldrich Co.) for histological evaluation. The specimens of the test and control groups were examined and photographed in the light microscope.

There is no standard histopathological scoring system in the literature to assess distal end-organ injury for I/R related myocardium. Following aortic cross-clamping, free oxygen radicals in the ischemic lower extremity during reperfusion, complement activation, and neutrophil chemotaxis are the main components of distal end-organ ischemia-reperfusion injury^[^^5^^,^^14^^]^. The main target of all these components is the endothelial barrier in the coronary microvascular region. An intraendothelial gap is created due to endothelial injury and myocardial edema, eventually^[^^15^^,^^16^^]^. With endothelial injury, the chemotaxis of polymorphonuclear leukocyte (PMNL) infiltration occurs^[^^5^^,^^14^^]^. In addition, activation of the complement system enhances tissue edema, directly leading to cardiomyocyte lysis^[^^5^^,^^17^^]^. Complete injury of the endothelium in the coronary microvascular region results in focal hemorrhages. Thus, the removed hearts were histopathologically investigated for myocytolysis, polymorphonuclear leukocyte infiltration, myofibrillar edema and focal hemorrhage.

## Statistical Analysis

Statistical analysis was performed using the PASW version 19.0 (SPSS Inc., Chicago, IL, USA) of the Department of Biostatistics, Faculty of Medicine of Trakya University (License No=10240642). Data were expressed in median (min-max) values or number and percentage. The Kruskal-Wallis test was used for intergroup data comparisons. The Bonferroni-corrected Mann-Whitney U test was used to detect groups which recorded a difference. Statistical significance was set at *P*<0.0167 for the Bonferroni-corrected Mann-Whitney U test. A *P* value of less than 0.05 was considered statistically significant.

## RESULTS

## Histopathological Examination

At the cellular level, no myocytolytic appearance was observed in all groups. However, only two samples in the I/R group (25%) were observed to develop light PMNL infiltration. Myofibrillar edema and focal hemorrhage were detected at a mild and moderate level. Data pertaining to microscopic examination of heart tissue sections of the test groups were compared using Kruskal-Wallis test. Comparison of the three groups produced no statistically significant difference in terms of myocytolysis (*P*=1.000) and PMNL infiltration (*P*=0.124), whereas there was a statistically significant difference with regards to myofibrillar edema (*P*=0.011) and focal hemorrhage (*P*<0.001) scores of the groups.

Histopathologic scoring was rated as +1: no change; +2: light injury; +3: moderate injury; +4: diffuse injury. Results for all the groups are shown in Table 1.

**Table 1:** The histopathological results of all test groups and the control group.

	Myocytolysis	PMNL infiltration	Myofibrillar edema	Focal hemorrhage
C-1	1	1	1	1
C-2	1	1	1	1
C-3	1	1	1	1
C-4	1	1	1	1
C-5	1	1	1	1
C-6	1	1	1	1
C-7	1	1	1	1
C-8	1	1	1	1
I/R -1	1	2	1	2
I/R -2	1	2	2	2
I/R -3	1	1	1	2
I/R -4	1	1	2	2
I/R -5	1	1	2	3
I/R -6	1	1	2	2
I/R -7	1	1	2	2
I/R -8	1	1	1	3
I/R +A-1	1	1	1	1
I/R +A -2	1	1	1	1
I/R +A -3	1	1	1	1
I/R +A -4	1	1	1	1
I/R +A -5	1	1	2	2
I/R +A -6	1	1	1	1
I/R +A -7	1	1	1	1
I/R +A -8	1	1	1	1

C=control; I/R=ischemia-reperfusion; I/R+A=ischemia-reperfusion + Acetaminophen; PMNL=polymorphonuclear leukocyte Histopathologic scoring was rated as +1 = no change; +2 = light injury; +3 = moderate injury; +4 = diffuse injury.

**Fig. 1:**
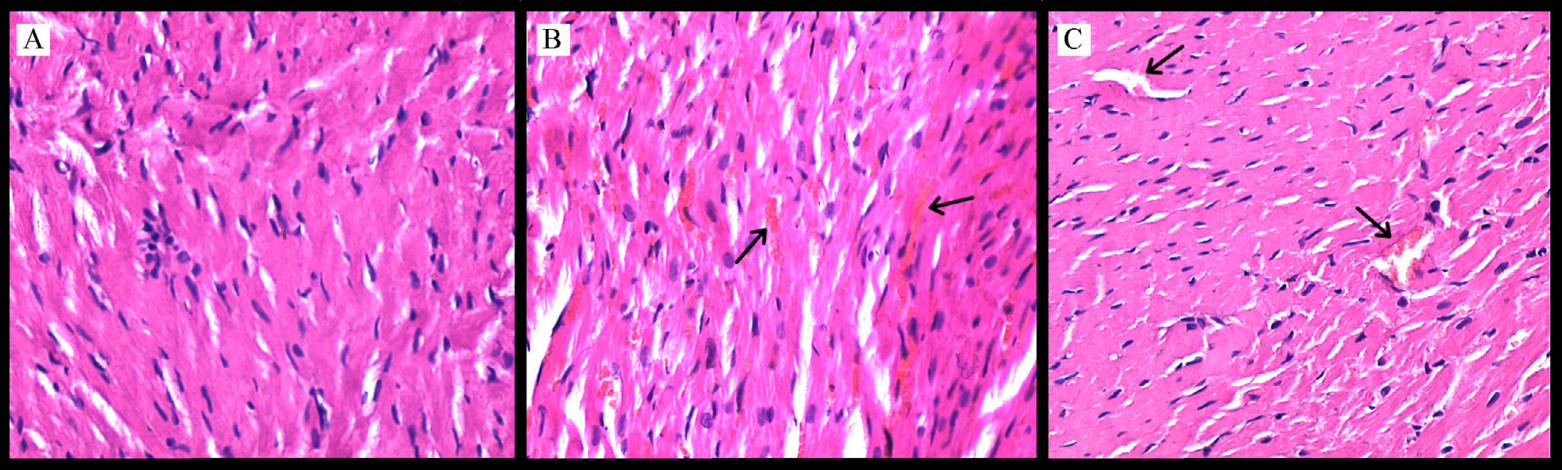
Acetaminophen attenuates the morphological changes associated with aortic I/R-induced myocardial injury. %" (A) In the control group, the normal appearance of cardiac myofibrillar. (B) In the aortic I/R group, changes in the myofibrillar swelling and thick focal hemorrhage. Erythrocytes are seen as evidence of an intense hemorrhage (black arrows) (C) In the aortic I/R + acetaminophen group, there is mild myofibrillar swelling and focal hemorrhage (blood vessels have a normal appearance) (black arrows). Heart sections are stained by hematoxylin and eosin, and examined by a light microscope. Magnification: original ×200; scale bar: 300 µm.

In the control group, histology of the myocardium was found to be normal and there were no changes in the myofibrillar appearance. However, myofibrillar swelling and thick focal hemorrhage were observed in the I/R group, and mild myofibrillar swelling in the I/R+A group (Figure 1).

Data pertaining to microscopic examination of heart tissue sections of the study groups were compared using the Kruskal-Wallis test. Comparison of the three groups demonstrated no statistically significant difference in terms of myocytolysis (*P*=1.000) and polymorphonuclear leukocyte infiltration (*P*=0.124), whereas myofibrillar edema (*P*=0.011) and focal hemorrhage (*P*<0.001) scores of the groups differed on a statistically significant level.

Group-related distributions of histopathological scores of myofibrillar edema and focal hemorrhage, which were detected using the Kruskal-Wallis test, revealed statistically significant differences between the study groups.

The Bonferroni-corrected Mann-Whitney U test was used to make statistical analysis of pair comparisons between the study groups. Statistical significance was set at *P*<0.0167. Myocytolysis and PMNL infiltration were not considered in the pair group comparisons since they were not found in heart tissue sections, and the Kruskal-Wallis test revealed no statistically significant difference.

Comparison of the control and I/R groups using Bonferroni-corrected Mann-Whitney U test revealed a statistically significant difference between the focal hemorrhage (*P*<0.001) and myofibrillar edema (*P*=0.009) scores of the two groups.

Comparison of the I/R and I/R + A groups using Bonferroni-corrected Mann-Whitney U test revealed a statistically significant difference between the focal hemorrhage (*P*=0.007) scores, while the difference between the same two groups in terms of myofibrillar edema (*P*=0.046) was found to be statistically insignificant (Table 2).

**Table 2:** Comparison of the groups regarding myofibrillar edema and focal hemorrhage.

	Myofibrillar edema	Focal hemorrhage
Control - I/R	*P*=0.009*	*P*<0.001*
Control - I/R +A	*P*=0.317	*P*=0.063
I/R - I/R +A	*P*=0.046	*P*=0.007*

Bonferroni-corrected Mann-Whitney U test,

* *P*<0.0167.

I/R=Ischemia-Reperfusion; I/R+A=Ischemia-Reperfusion +Acetaminophen

## DISCUSSION

Results of the present study demonstrate that, acetaminophen can reduce myocardial injury in rats, induced by the I/R which is caused by abdominal aortic cross-clamping. Histopathological examinations showed that the acetaminophen administered group suffered less from focal hemorrhage and myofibrillar edema induced by I/R injury in rats, which supports the suggestion of this study. No statistically significant difference was detected between the two groups in terms of myofibrillar edema; however, myofibrillar edema was observed to be suffered less by the acetaminophen + I/R group.

Aortic cross-clamping inserted during abdominal aortic aneurysm operation, induces ischemia in the lower extremity. Reperfusion of the ischemic leg with oxygenized blood results in the formation of toxic oxygen metabolites, leukocyte activation, cytokine release and activation of the complement cascade^[^^17^^]^. I/R injury is known to develop in organs such as the lungs, liver and kidney due to oxidative stress and inflammatory response caused by high amount of reactive oxygen products produced by lower extremity reperfusion after the removal of cross-clamping^[^^5^^,^^14^^]^. Hydrogen peroxide, hydroxyl and superoxide radicals, the main reactive oxygen products, are shown to result in peroxidation in membrane phospholipids of myocardial tissues cells^[^^4^^]^. In addition, I/R injury is suggested to increase by remote organ infiltration of activated neutrophils due to aortic I/R and the myeloperoxidase enzyme released by them^[^^4^^,^^18^^,^^19^^]^. Moreover, released cytokines, particularly tumor necrosis factor-, induce leukocyte activation and also damage capillary continuity^[^^17^^]^. Formation of membrane attack complex, the final product of the complement cascade due to activation of the complement cascade, contributes to damage the integrity of the cell membrane. C3a and C5a, which are products of this cascade and known as anaphylatoxins, increase leukocyte chemotaxis^[^^17^^]^. As understood, distant organ I/R injury is not a single reaction, but rather a complex process with intracellular and extracellular pathophysiologic changes. Histological changes in the heart, resulting from I/R, can be seen via the light microscope^[^^19^^]^. Variables such as myofibrillar edema and focal hemorrhage detected under the light microscope were recorded in the I/R group, but not in the control group, which is suggested to be an indicator of distant organ I/R injury in the heart due to abdominal aorta operation.

When a radical catcher is used to decrease reperfusion injury, considering the fact that free radicals rapidly develop within a few minutes after reperfusion, the catcher should be used just before the ischemic period starts or 15 minutes before reperfusion since it has no preventive effect if used after reperfusion^[^^20^^]^. As a result, in the scope of the present study, APAP infusion started 15 minutes before aortic cross-clamping removal.

There are discussions on the mechanism of action of APAP, one of the most popular and common drugs used in the treatment of pain and temperature. The drug continues to be a mystery of pharmacology due to its unknown therapeutic effects^[^^21^^]^. There are experimental studies showing the possible effects of APAP on reduction of ischemia-reperfusion injury^[^^11^^-^^13^^,^^22^^-^^30^^]^. Different studies offer different mechanisms in explaining the effect of APAP. An experimental study by Hadzimichalis et al.^[^^22^^]^ showed that one of the main mechanisms of I/Rinjury is the opening of mitochondrial permeability transition pore and the increase in mitochondrial cytochrome release, and that APAP reduces I/R injury and induces myocyte apoptosis by preventing this opening and increase. In a study by Baliga et al.^[^^23^^]^, acetaminophen was shown to prevent mitochondrial dysfunction thanks to a similar affect it creates in I/R in rat brains. In another experimental study, Merrill^[^^24^^]^ revealed that APAP, some phenols of which have antioxidant characteristics, is a phenol compound, and in turn prevents the release of hydroxyl radicals, which develop in high amounts in the first minutes of reperfusion, and also reduces peroxide production. In an experimental study by Rork et al.^[^^25^^]^, APAP was shown to have cardioprotective effects by reducing I/R injury by preventing destruction of matrix metalloproteinase-2-induced troponin-I activated by peroxynitrite. Many studies were conducted according to different systems, showing the antioxidant characteristics of APAP^[^^26^^-^^28^^]^. These studies also showed that APAP reduces *in-vivo* and *in-vitro* mitochondria-induced reactive oxygen amount and suggest that APAP does it by acting like α-tocopherol and like a direct phenolic radical scavenger. In addition, Nam et al.^[^^30^^]^ showed in their study that acetaminophen reacts with peroxyl radicals more than with other phenol compounds used as antioxidant. In their study, Jaques-Robinson et al.^[^^31^^]^ showed that APAP is a cardioprotective and antiarrhythmic agent against the oxidative damage induced by hydrogen peroxide in canine heart.

Different studies offer different durations for I/R in the development of myocardial damage after lower extremity I/R. In a study by Kiris et al.^[^^4^^]^ in which the rats were induced with 30 min ischemia followed by 60 min reperfusion, malonyldialdehyde, catalase, superoxide dismutase levels, and myeloperoxidase activity were found to be statistically, significantly higher in the aortic I/R group compared to the control group. In the aforementioned study, the authors found that the heart was biochemically affected by elevated oxidative stress metabolism biomarkers with 30 min ischemia and 60 min reperfusion^[^^4^^]^. In their study where 120 minutes of ischemia were followed by 120 minutes of reperfusion, Narin et al.^[^^19^^]^ found statistically significant higher malonyldialdehyde, catalase, and superoxide dismutase levels in the aortic I/R group compared to the control group. In the histopathological examination, myocardial disorganization, myocardial swelling, and myofiber eosinophilia were examined and all these variables were found to be statistically, significantly higher in the ischemia-reperfusion group. However, Koçarslan et al.^[^^7^^]^ performed 45 min ischemia and 60 min reperfusion and found no significant difference in the I/R injury using cardiac biochemical or histopathological parameters. In an experimental study, Aydin et al.^[^^8^^]^ performed 60 min ischemia and 120 min reperfusion, as in our study. Similar to Koçarslan et al.^[^^7^^]^'s study, the authors examined interstitial edema, inflammatory cellular infiltration, and coagulation necrosis, and they were able to find a significant difference only in the myofibrillar swelling in the I/R group. Koçarslan et al.^[^^7^^]^ showed that, although the biomarkers of the oxidative stress metabolism increased during I/R, there was no histopathological sign indicating that 45 min ischemia does not lead to cardiac injury as assessed by light microscopy. These findings suggest that 60 min ischemia at least should be applied for distal end-organ injury in the lower extremity I/R model in rats, as in our study. Although PMNL is a component for I/R injury, it did not lead to a statistically significant difference in cardiac infiltration in our study and previous studies. Although we found a significant difference in myofibrillar swelling and focal hemorrhage between the control and ischemia-reperfusion groups, we observed no myocytolysis and PMNL infiltration, indicating no severe reaction to achieve enhanced complement system activation and significant PMNL infiltration in the I/R model (Table 2). Therefore, we suggest longer I/R duration to histopathologically examine these four parameters under light microscopy.

The present study suggests that APAP reduced focal hemorrhage in the I/R group at a statistically significant level but had no such effect on myofibrillar edema. The statistical significance of our study was increased by lowering the statistical significance down to *P*<0.0167. As a result of the Bonferroni correction, the examination of median values shows that myofibrillar edema was histopathologically higher in the I/R group, although acetaminophen was observed not to reduce myofibrillar edema at a statistically significant level (Table 2). Authors of the present study suggest that despite having no statistically significant preventive effect on myofibrillar edema, acetaminophen has a clinically protective effect.

There are two limitations of the present study. The first limitation is the small sample size, while the second limitation is that cardiac injury resulting from ischemia-reperfusion injury and reactive oxygen metabolite activity induced by I/R were not analyzed using biochemical indicators.

## CONCLUSION

In conclusion, acetaminophen is considered to have cardioprotective effects by reducing myocardial injury induced by abdominal aortic ischemia-reperfusion. Studies continue to be made to reduce ischemia-reperfusion injury in times when cardiac morbidity and mortality is on the increase. Future clinical studies should be made concerning the effect of acetaminophen.

**Table:** 

Authors' roles & responsibilities	
OG	Substantial contributions to the conception or design of the work; the acquisition, analysis, or interpretation of data for the work; drafting the work or revising it critically for important intellectual content; agreement to be accountable for all aspects of the work in ensuring that questions related to the accuracy or integrity of any part of the work are appropriately investigated and resolved; final approval of the version to be published
EK	Drafting the work or revising it critically for important intellectual content; agreement to be accountable for all aspects of the work in ensuring that questions related to the accuracy or integrity of any part of the work are appropriately investigated and resolved; final approval of the version to be published
CSÜ	Substantial contributions to the conception or design of the work; the acquisition, analysis, or interpretation of data for the work; final approval of the version to be published
SC	Substantial contributions to the conception or design of the work; the acquisition, analysis, or interpretation of data for the work; drafting the work or revising it critically for important intellectual content; final approval of the version to be published
